# Risk factors for healthcare-associated infections and their relationship with waves of the COVID-19 pandemic in an intensive care unit: a nested case-control study

**DOI:** 10.31744/einstein_journal/2024AO0939

**Published:** 2024-11-26

**Authors:** Fernando Gatti de Menezes, Thiago Domingos Corrêa, Bruno de Arruda Bravim, Paula Tuma, Moacyr Silva, Emy Akiyama Gouveia, Alexandra do Rosário Toniolo, Graziela Geanfrancisco Matta de Paiva, Paula Fernanda Martineli, Helena Maria Fernandes Castagna, Talita Silva Sarro Moraes, Ana Carolina Santiago, Priscila Gonçalves, Brunna Oliveira Pereira, Nathalia Thomazi Gonçalves, Daniel Tavares Malheiro, Vanessa Damazio Teich, Miguel Cendoroglo

**Affiliations:** 1 Hospital Israelita Albert Einstein São Paulo SP Brazil Hospital Israelita Albert Einstein, São Paulo, SP, Brazil.

**Keywords:** COVID-19, Coronavirus infections, Risk factor, Pneumonia, ventilator-associated, Catheter-related infections, Intensive care unit, Pandemics

## Abstract

**Objective:**

To evaluate the risk factors for healthcare-related infections during the COVID-19 pandemic in intensive care units, to investigate the impact of COVID-19 on Central Line-Associated Bloodstream Infection, Catheter-Associated Urinary Tract Infection, and ventilator-associated pneumonia, and to describe healthcare-associated infections in the waves of the COVID-19 pandemic.

**Methods:**

This nested case-control study was conducted in a 137-bed adult medical/surgical intensive care unit at a private hospital in São Paulo, Brazil, between January 11, 2019, and May 21, 2022. Case patients were identified using the Nosocomial Infection Control Committee database and control patients were identified using the intensive care unit's EPIMED system. For the analysis of risk factors, the chi-square test, multiple logistic regression model, and Kaplan-Meier method were used to identify independent risk factors, considering p<0.05.

**Results:**

The Case Group consisted of 189 healthcare-associated infections, including ventilator-associated pneumonia (61.4%), Central Line-Associated Bloodstream Infection (30.1%), Catheter-Associated Urinary Tract Infection (8.5%), and the Control Group consisted of 6,834 patients. The independent risk factors were COVID-19 infection (OR=2.84, 95%CI=1.92–4.23, p<0.01), length of stay in intensive care greater than 14 days (OR=3.15, 95%CI=1.95–5.14, p<0.01), length of hospital stay greater than 30 days (OR=3.64, 95%CI=2.44–5.51, p<0.01), and patients who were in the third wave (OR=1.72, 95%CI=1.05–2.91, p=0.04). Ventilator-associated pneumonia was the only healthcare-related infection for which COVID-19 infection was an independent risk factor (OR=3.32, 95%CI=1.92–5.94, p<0.01).

**Conclusion:**

COVID-19 and length of hospital stay were independent risk factors for healthcare-associated infections, and only ventilator-associated pneumonia was affected by the COVID-19 pandemic.

## In Brief

Menezes et al. described their nested case-control study conducted between 2019 and 2022 in a private hospital regarding the risk factors of healthcare-related infections during the COVID-19 pandemic. COVID-19 and length of hospital stay were the most important risk factors, and ventilator-associated pneumonia was the only healthcare-related infection for which COVID-19 was an independent risk factor.

## Highlights

■ Healthcare-associated infections are responsible for higher morbidity and mortality rates.
■ The COVID-19 pandemic brought a scenario of enormous demand for healthcare.
■ Knowledge of risk factors is important for preventive measures.
■ COVID-19 and the length of hospital stay were the most important risk factors for healthcare-associated infections.

## Graphical abstract

**Figure:**
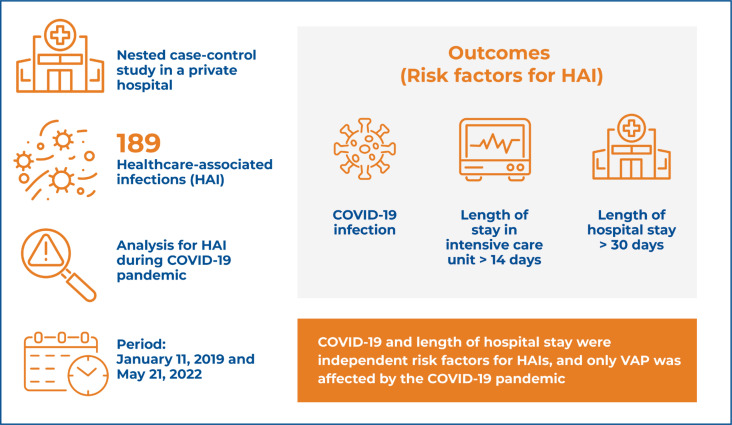

## INTRODUCTION

Healthcare-associated infections (HAIs) are responsible for increased length of hospital stay, cost of hospitalization, and high morbidity and mortality.^(1)^ Before the COVID-19 pandemic, literature suggested that up to 55% of HAIs could be prevented using effective prevention strategies. However, the COVID-19 pandemic has brought enormous demand for healthcare, work overload, shortage of personal protective equipment, reduction of the health workforce due to absenteeism, and reduction in the quality of the best prevention practices in HAI control. Furthermore, the immunosuppressive treatments used for SARS-CoV-2 infection, such as corticosteroids and interleukin-6 inhibitors, made more patients susceptible to infections similar to HAI.^(2–4)^

Several studies have verified that COVID-19 is a risk factor for HAIs, increasing the incidence of central line-associated bloodstream infections, catheter-associated urinary tract infections, and ventilator-associated pneumonia.^(5–7)^ However, few studies have been conducted on the impact of COVID-19 waves on the incidence of HAI.^(8)^

## OBJECTIVE

Thus, this retrospective study evaluated the risk factors for healthcare-associated infections during the COVID-19 pandemic in an intensive care unit including the impact of waves of the COVID-19 pandemic.

## METHODS

This retrospective study was conducted in a 137-bed adult medical/surgical intensive care unit (ICU) and step-down unit in a private hospital in the city of São Paulo, Brazil, and was divided into four periods of analysis (before the COVID-19 pandemic: January 11, 2019, to February 22, 2020; first wave of the COVID-19 pandemic: February 23, 2020, to July 25, 2020; second wave of the COVID-19 pandemic: July 26, 2020, to April 10, 2021; third wave of the COVID-19 pandemic: April 11, 2021, to May 21, 2022). The wave intervals were determined by the Brazilian Ministry of Health.^(9)^ The Case Group consisted of individuals who developed HAIs, whereas the Case Group consisted of individuals who did not develop HAI. The exclusion criteria were patients aged <18 years, and lack of evidence of HAI before the beginning of our study.

Healthcare-associated infection diagnostic criteria followed the annual guidelines of the Centers for Disease Control and Prevention (CDC).^(10)^ Information on the Case Groups was obtained from the Nosocomial Infection Control Committee database. The following variables were evaluated between the Case and Control Groups: age, gender, body mass index (BMI), pre-pandemic periods and pandemic waves, severity scores upon patients’ admission to the ICU without reassessment throughout the study, simplified acute physiology score (SAPS) 3 and Charlson index, COVID-19 infection, length of stay in the intensive care unit (≤ 14 days or >14 days), and length of hospital stay (≤ 30 days or >30 days) before the HAI event. All data were obtained from the EPIMED system of the ICU.^(11)^ In the univariate analysis, the χ^2^ test was used; in the multivariate analysis, including the variables in the univariate analysis with p<0.1, the multiple logistic regression model was used to identify independent risk factors, considering p<0.05.

Finally, we analyzed survival without healthcare-related infections using the Kaplan-Meier method (Cox regression) to assess the impact of COVID-19. Statistical analyses were performed using R software version 4.4.1 (R Foundation for Statistical Computing, Vienna, Austria) and IBM SPSS Statistics 23.^(12)^

This study was approved by the Research Ethics Committee of *Hospital Israelita Albert Einstein* Hospital under protocol numbers CAAE: 58453422.7.0000.0071; #5.605.087.

## RESULTS

During the study period, the Case Group consisted of 189 HAIs, including ventilator-associated pneumonia (VAP) (61.4%), Central Line-Associated Bloodstream Infection (CLABSI) (30.1%), and Catheter-Associated Urinary Tract Infection (CAUTI) (8.5%), while the Case Group consisted of 6,834 patients without HAI.

In table 1, the independent risk factors for acquiring HAI were: COVID-19 infection (OR=2.84, 95%CI=1.92–4.23, p<0.01), length of stay in intensive care greater than 14 days (OR= 3.15, 95%CI=1.95–5.14, p<0.01), length of hospital stay greater than 30 days (OR= 3.64, 95%CI=2.44–5.51, p<0.01). The survival curve without healthcare-related infections in patients with COVID-19 over the length of hospital stay was worse than that in patients without COVID-19 (Log-rank Mantel-Cox 8.792, p=0.003).

**Table 1:** Risk factors for acquiring healthcare-associated infections during the pandemic

Characteristics		Case (n=189) n (%)	Control (n=6,834) n (%)	Univariate analysis		Multivariate analysis	
				OR (95%CI)	p value	OR (95%CI)	p value
Age							
	≤60 years	70 (37)	2,243 (32.8)	-	-	-	-
	>60 years	119 (63)	4,591 (67.2)	0.83 (0.62–1.13)	0.22	0.71 (0.49–1.03)	0.08
Gender							
	Male	138 (73)	3,856 (56.4)	2.09 (1.52–2.92)	<0.01	1.40 (1–1.99)	0.06
	Female	51 (27)	2,978 (43.6)	-	-	-	-
BMI							
	BMI≤30	127 (67.2)	5,209 (76.2)	-	-	-	-
	BMI>30	62 (32.8)	1,625 (23.8)	1.56 (1.14–2.12)	<0.01	1.18 (0.84–1.65)	0.33
Before the COVID-19 pandemic		23 (12.2)	2,345 (34.3)	-	-	-	-
First wave of COVID-19 pandemic		22 (11.6)	617 (9)	3.64 (2.00–6.58)	<0.01	1.26 (0.65–2.42)	0.49
Second wave of COVID-19 pandemic		62 (32.8)	1,414 (20.7)	4.47 (2.80–7.39)	<0.01	1.34 (0.77–2.39)	0.31
Third wave of COVID-19 pandemic		82 (43.4)	2,458 (36)	3.40 (2.17–5.54)	<0.01	1.72 (1.05–2.91)	0.04
SAPS 3							
	Score≤49	101 (53.4)	4,518 (66.1)	-	-	-	-
	Score>49	88 (46.6)	2,316 (33.9)	1.70 (1.27–2.27)	<0.01	1.08 (0.74–1.57)	0.69
Charlson index							
	≤3	159 (84.1)	5,656 (82.8)	-	-	-	-
	>3	30 (15.9)	1,178 (17.2)	0.91 (0.60–1.32)	0.62	0.83 (0.52–1.29)	0.43
COVID-19 infection							
	Yes	105 (55.6)	834 (12.2)	8.99 (6.70–12.1)	<0.01	2.84 (1.92–4.23)	<0.01
	No	84 (44.4)	6,000 (87.8)	-	-	-	-
Length of stay in the intensive care unit							
	≤14 days	38 (20.1)	5,313 (77.7)	-	-	-	-
	>14 days	151 (79.9)	1,521 (22.3)	13.9 (9.79–20.2)	<0.01	3.15 (1.95–5.14)	<0.01
Length of hospital stay							
	≤30 days	58 (30.7)	5,685 (83.2)	-	-	-	-
	>30 days	131 (69.3)	1,149 (16.8)	11.2 (8.19–15.4)	<0.01	3.64 (2.44–5.51)	<0.01

BMI: body mass index.

In relation to the waves of the pandemic (Table 1), patients who were in the third wave had a higher risk of acquiring HAIs than those in other periods (OR=1.72, 95%CI=1.05–2.91, p=0.04).

In table 2, VAP was the only healthcare-related infection for which COVID-19 infection was an independent risk factor (OR=3.32, 95%CI=1.92–5.94, p<0.01).

**Table 2:** Impact of COVID-19 infection on CLABSI, CAUTI, VAP

	CLABSI		Univariate analysis		Multivariate analysis	
	Yes, n (%)	No, n (%)	OR (95%CI)	p value	OR (95%CI)	p value
COVID-19 infection, Yes	21 (36.8)	631 (17.5)	2.75 (1.57–4.70)	<0.01	1.11 (0.53–2.28)	0.79
COVID-19 infection, No	36 (63.2)	2.973 (82.5)	-	-	-	-
	**CAUTI**					
	**Yes, n (%)**	**No, n (%)**				
COVID-19 infection, Yes	4 (25)	721 (12.3)	2.38 (0.66–6.84)	0.13	1.10 (0.26–4.16)	0.89
COVID-19 infection, No	12 (75)	5.139 (87.7)	-	-	-	-
	**VAP**					
	**Yes, n (%)**	**No, n (%)**				
COVID-19 infection, Yes	80 (69)	640 (30.3)	5.11 (3.44–7.74)	<0.01	3.32 (1.92–5.94)	<0.01
COVID-19 infection, No	36 (31)	1.473 (69.7)	-	-	-	-

## DISCUSSION

The main HAI in patients with COVID-19 was VAP, accounting for 61.4% of HAIs in this study, while other studies reported VAP as 50–67% of HAIs.^(13,14)^ The main reason for this is related to the nature of pulmonary involvement in COVID-19 and its severity.

Regarding risk factors for HAI, this study demonstrated that COVID-19 was an independent variable with OR=2.84, 95%CI=1.92–4.23, p<0.01. Another study also proved the importance of COVID-19 with adjusted hazards ratio 1.65, 95%CI=1.38–1.96, p<0.05.^(15)^ The main cause of this was the longer length of stay in the intensive care unit and hospital, associated with the complexity of the management of COVID-19, which was also found to be an independent risk factor in this study, similar to other studies.^(16,17)^

As described in another study, a long stay in intensive care provides greater exposure to invasive devices such as indwelling bladder catheters, central venous catheters, and endotracheal tubes, which are related to the main HAIs: CLABSI, CAUTI, and VAP.^(17)^ One study demonstrated a 3.7-fold increase in the incidence of CLABSI and a 2.7-fold increase in CAUTI in patients with COVID-19, consistent with the findings of other studies.^(18,19)^ However, this study only demonstrated the impact of COVID-19 as an independent risk factor for VAP, (OR=3.32, 95%CI=1.92–5.94, p<0.01) and not for CLABSI (OR=1.11, 95%CI=0.53–2.28, p=0.79) and CAUTI (OR=1.10, 95%CI=0.26–4.16, p=0.89) (Table 2). A possible explanation for the lack of evidence in this study on the impact of COVID-19 on CLABSI and CAUTI is related to the small sample size (CLABSI, n=57; CAUTI, n=19) compared with that of VAP (n=116).

An interesting finding from the study was the detection of a higher risk for HAI in the third wave of the pandemic (OR=1.72, 95%CI=1.05–2.91, p=0.04). This may be explained by the fact that no outbreaks occurred during the third wave. Studies on the impact of COVID-19 waves on HAIs are not comparable because of the different wave timing classifications in different countries.^(18,19)^ The possible explanation for this evidence is related to the duration of each wave, with the first wave lasting approximately 5 months (February 23, 2020 to July 25, 2020), the second wave lasting approximately 8 months (July 26, 2020 to April 10, 2021), and the third wave lasting approximately 13 months (April 11, 2021 to May 21, 2022).

A limitation of this study is that its retrospective design may have resulted in incomplete information from the medical records. Second, unlike the VAP, the sample sizes for CLABSI and CAUTI influenced the analysis of the impact of COVID-19. Third, it was not possible to compare the waves between publications because of the different intervals in each country. Fourth, this retrospective study did not use case-control pairing; however, the analysis did not demonstrate differences between age, sex, and other variables, such as severity (Charlson index and SAPS3) which could interfere with the analysis between the Case and Control Groups.

## CONCLUSION

COVID-19 is an independent risk factor for healthcare-associated infections, particularly ventilator-associated pneumonia, but not Central Line-Associated Bloodstream Infection or Catheter-Associated Urinary Tract Infection. The lengths of hospital stay and intensive care were also risk factors for healthcare-associated infections. The scarcity of studies on the relationship between healthcare-associated infections infections and waves of the COVID-19 pandemic makes it important to conduct more studies on this subject.
